# Simultaneous Determination of Gross Alpha/Beta Activities in Groundwater for Ingestion Effective Dose and its Associated Public Health Risk Prevention

**DOI:** 10.1038/s41598-020-61203-y

**Published:** 2020-03-09

**Authors:** Phan Long Ho, Le Dinh Hung, Vu Tuan Minh, Dang Van Chinh, Tran Thien Thanh, Chau Van Tao

**Affiliations:** 1 Department of Nuclear Physics, Faculty of Physics and Engineering Physics, University of Science, Ho Chi Minh City, Vietnam; 2 Vietnam National University, Ho Chi Minh City, Vietnam; 3 Nuclear Technique Laboratory, University of Science, Ho Chi Minh City, Vietnam; 4 Institute of Public Health in Ho Chi Minh City, Ho Chi Minh City, Vietnam

**Keywords:** Environmental monitoring, Risk factors, Atomic and molecular interactions with photons

## Abstract

This paper presents information on the gross alpha and gross beta activity concentrations of two hundred twenty-six groundwater samples collected by gas flow proportional counters in southern Vietnam. The gross alpha results in the water samples ranged from 0.024 to 0.748 Bq L^−1^ with a mean of 0.183 ± 0.034 Bq L^−1^, and the gross beta results in the water samples ranged from 0.027–0.632 Bq L^−1^ with a mean of 0.152 ± 0.015 Bq L^−1^. The values obtained in this work were compared with those previously published for various regions or countries. Next, untreated and treated groundwater samples were analyzed to assess their influences on the treatment process. The results showed that there were differences in the minimum detection concentrations and the mean activity values between the untreated and treated groundwater samples (The p-value of the mean comparison tests is significant with p < 0.05). In both sample groups, there was a strong positive correlation of the gross alpha versus the gross beta results (r > 0.6). This means that among the radionuclides, the major sources of beta radiation are uranium and thorium decay series radionuclides. Finally, the annual effective dose for adults (>17 years) was calculated based on the assumption that major radionuclides have the highest effective dose conversion factors. In general, the results for Pb-210, Ra-226, and Ra-228 were observed to be lower than the recommended reference values established by the World Health Organization and the International Atomic Energy Agency, except for the value of Po-210.

## Introduction

Water is the most important substance in our lives, and the management of water resources is a national and international problem. Water is an essential and basic human right and protecting the quality of water is also a component of effective health policy^1–4^. Water is considered to be a factor that can increase the exposure of humans to natural radiation^5^. The radioactivity present in groundwater may be mainly from the following radionuclides: nuclides in the U-238 and Th-232 decay series, K-40, cosmic rays, authorized discharges from nuclear facilities and other licensed facilities, fallout from nuclear weapon tests, and accidental releases of radionuclides^4^. The primary alpha-emitting radionuclides in the natural decay series are U-238 and Th-232 and its progenies U-234, Th-230, Ra-226, Po-210, and Th-228. In general, gross beta activity concentrations^6–9^ are primarily due to K-40, Ra-228, and Pb-210.

There are several standards or regulations limiting concentrations of radionuclides in the water samples, which requires the ability to identify and quantify radionuclides utilizing different methods, such as alpha spectroscopy^10–13^, gamma spectroscopy^13–16^, and liquid scintillation counting^17,18^. However, the process of identifying separation procedures for radionuclide concentrations in water samples is time consuming and expensive. Therefore, the simplest practical approach is to use a screening method based on the measurement of gross alpha and gross beta activity concentrations without regard to the identity of specific radionuclides^2,3,9,19^. This screening method is also the first step of radiological characterization in the field of radioecology, environmental monitoring, and industrial applications. Its major advantages are its low cost and simplicity^9,20^.

In this work, the gross alpha and gross beta activity concentrations of two hundred twenty-six groundwater samples in southern Vietnam were determined from untreated and treated samples. The annual effective doses of groundwater ingestion for the adult age group were evaluated and compared to the reference dose recommended for radionuclides in drinking water. To date, no study has been performed in this region on groundwater radioactivity.

## Materials and Methods

### Study area and water sampling

Southern Vietnam includes southeast and Mekong River delta regions^21^. The nation measures approximately 64,369 km^2^ and has a population of approximately 34.48 million, as of 2017. The samples were collected by the partners of the Institute of Public Health in Ho Chi Minh City. In all 226 samples, 108 samples (labeled S1-S108) have untreated groundwater as the source with a mean total dissolved solids (TDS) content of 255 ± 134 mg L^−1^, and the other samples (S109-S226) have treated groundwater as the source with a mean TDS content of 124 ± 113 mg L^−1^. In which, the untreated sample was collected directly from drilling well and the treated sample was taken from the filtration system with technologies such as filter, ion exchange, reverse osmosis, etc. The samples were collected in 1-liter plastic containers, and then the samples were acidified with nitric acid (HNO_3_) until reaching a pH level below 2 to avoid any biological activity and adsorption losses of radionuclides around the container walls and on solid surfaces.

### Determination of gross alpha and beta activities by GFPC

The gross alpha and gross beta activity measurements, called the thin source deposit method, were performed as the first step in the radiological characterization of the water samples, and this method includes the standards ISO 10704:2009 for non-saline water^22^. This procedure is accredited as ISO 17025:2017 by the Vietnam Bureau of Accreditation. For the gross alpha and gross beta analyses, 250 ml of each groundwater sample was evaporated without boiling at ≤85 °C (ensuring deposits with a surface density below 5 mg cm^−2^). The obtained residue was transferred to a stainless steel planchet (2 inch diameter and 1/8 inch depth). Each planchet was measured for gross alpha and gross beta activity during a 120 min interval per sample at the Institute of Public Health in Ho Chi Minh City using a low-background WPC-1050 (Protean Instruments Corporation). The detector type was a gas flow proportional counter (GFPC) with a mixture of 90% argon and 10% methane (P-10) and an automatic 50 sample transport. The operating high voltage of the detector was set at 1,515 V. The background of each detector was determined by counting empty planchets for 3,600 min.

The detectors were calibrated for alpha and beta efficiencies using Am-241 (10.0 ± 0.1 Bq) and Sr-90/Y-90 (10.3 ± 0.2 Bq) standard solution sources, which were supplied by the Eckert & Zeigler company. The gross alpha efficiency was 18.4 ± 0.4%, while the gross beta efficiency was 67.5 ± 0.5%, and the alpha-beta crosstalk correction factor (χ) was 32.1 ± 1.0%. The self-absorption curve of gross alpha activity concentration was made by adding the same Am-241 (3.53 ± 0.05 Bq) to seven tap water samples with different volumes of 0, 100, 200, 400, 600, 800, and 1000 mL. The solutions were prepared with the same analyzed sample preparation procedure; their residues were deposited on a planchet and were determined to be in the range of 0–100 mg. Then, they were counted by WPC-1050, and the self-absorption curves were fitted to the exponential function f_aα_ = 0.9986 × exp(−0.0097 × m), where m is the mass of the deposit (mg).

The gross alpha and gross beta activity concentrations can be obtained as followed^22^:

1 $${{\rm{A}}}_{{\rm{\alpha }}}({\rm{Bq}}\,{{\rm{L}}}^{-1})=\frac{{{\rm{r}}}_{{\rm{g}}{\rm{\alpha }}}-{{\rm{r}}}_{0\alpha }}{{\rm{V}}\times {\varepsilon }_{{\rm{\alpha }}}\times {{\rm{f}}}_{{\rm{a}}{\rm{\alpha }}}}$$

2 $${{\rm{A}}}_{{\rm{\beta }}}({\rm{Bq}}\,{{\rm{L}}}^{-1})=\frac{{{\rm{r}}}_{{\rm{g}}{\rm{\beta }}}-{{\rm{r}}}_{0{\rm{\beta }}}-{\rm{\chi }}({{\rm{r}}}_{{\rm{g}}{\rm{\alpha }}}-{{\rm{r}}}_{0{\rm{\alpha }}})}{{\rm{V}}{\times }_{\varepsilon {\rm{\beta }}}}$$

where A_α/β_ is the gross alpha/beta activity concentration of the sample (Bq L^−1^); V is the volume of the sample (L); ε_α_ and ε_β_ are the alpha and beta efficiencies, respectively; f_aα_ is the alpha self-absorption factor; the self-absorption of beta phenomena was negligible (f_aβ_ = 1); r_gα_ and r_gβ_ are the gross count rates from the alpha and beta windows, respectively (cps); r_0α_ and r_0β_ is the background count rate from the alpha and beta windows, respectively (cps); and χ is the crosstalk alpha-beta factor.

The minimum detectable concentration (MDC) for the gross alpha and gross beta activity concentration are calculated as follows^22,23^:

3 $${{\rm{MDC}}}_{{\rm{\alpha }}/{\rm{\beta }}}({\rm{Bq}}\,{{\rm{L}}}^{-1})=\frac{2{{\rm{c}}}_{{\rm{\alpha }}/{\rm{\beta }}}^{\ast }+({{\rm{k}}}^{2}{{\rm{w}}}_{{\rm{\alpha }}/{\rm{\beta }}}/{{\rm{t}}}_{{\rm{g}}})}{1-{{\rm{k}}}^{2}{{\rm{u}}}_{{\rm{rel}}}^{2}({{\rm{w}}}_{{\rm{\alpha }}/{\rm{\beta }}})}$$

where c^*^ is the decision threshold for the gross alpha and gross beta; k = 1.65; t_g_ is the sample counting time; u_rel_ is the relative standard uncertainty; and ${{\rm{w}}}_{{\rm{\alpha }}}=\frac{1}{{\rm{V}}\times {{\rm{f}}}_{{\rm{a}}\alpha }\times {{\rm{\varepsilon }}}_{\alpha }}$ and ${{\rm{w}}}_{{\rm{\beta }}}=\frac{1}{{\rm{V}}\times {{\rm{\varepsilon }}}_{{\rm{\beta }}}}.$

### Estimation of the annual effective dose

The annual effective dose (AED) for the gross alpha and gross beta activity concentrations associated with radiation exposure through ingestion of the groundwater samples was estimated to assess health risks to adult members of the public using the following equation^3^:

4 $${{\rm{AED}}}_{{\rm{\alpha }}/{\rm{\beta }}}({\rm{mSv}}\,{{\rm{y}}}^{-1})={{\rm{A}}}_{{\rm{\alpha }}/{\rm{\beta }}}\times {{\rm{IR}}}_{{\rm{w}}}\times {\rm{CF}}$$

where A_α/β_ is the gross alpha/beta activity concentration of the sample (Bq L^−1^); CF is the age-dependent effective dose conversion factor; and IR_w_ is the annual ingested volume of drinking water per year. In this work, IR_w_ = 730 L y^−1^ according to WHO and IAEA^3,4^ for an adult person and CF^24^ values for the main radionuclides was CF_Po-210_ = 1.2 × 10^−3^ mSv Bq^−1^, CF_Ra-226_ = 2.8 × 10^−4^ mSv Bq^−1^, CF_Pb-210_ = 6.9 × 10^−4^ mSv Bq^−1^, and CF_Ra-228_ = 6.9 × 10^−4^ mSv Bq^−1^.

The radiological criteria for groundwater quality were determined by the Ministry of Natural Resources & Environment and Ministry of Health published in the Vietnam National Technical Regulation. Moreover, the maximum admitted activity concentrations are 0.1 Bq L^−1^ for the gross alpha and 1 Bq L^−1^ for the gross beta^1,2^. These values are 0.5 Bq L^−1^ for the gross alpha activity concentration and 1 Bq L^−1^ for the gross beta activity concentration according to WHO and IAEA^3,4^. However, the annual effective dose value is equal to the WHO and IAEA recommended reference value^3,4^ of 0.1 mSv y^−1^.

## Results and discussions

### Validation of analytical procedure

Validation tests for the analytical procedure were carefully prepared by adding the spiked SRM-NIST 4322 C (Am-241) in water in the range of 0.05 Bq L^−1^ to 1.0 Bq L^−1^ for determination of the gross alpha and by adding the spiked NIST 4239 (Sr-90/Y-90) in water in the range of 0.5 Bq L^−1^ to 8.0 Bq L^−1^ for determination of the gross beta. The solutions were prepared according to the same sample analysis preparation procedure and counted by WPC-1050. For the gross alpha, the obtained recoveries, relative standard deviation and linearity between the measured and expected activity were from 94–98%, less than 7.5% and y_α_ = 0.9593x_α_ + 0.005 (R² = 0.9995; p < 0.001), respectively. Similarly, for the gross beta determination, the obtained recoveries were 86–94%, the relative standard deviation was 3.2%, and the linearity was y_β_ = 0.9871x_β_ − 0.0724 (R^2^ = 0.9996; p < 0.001).

**Figure 1:**
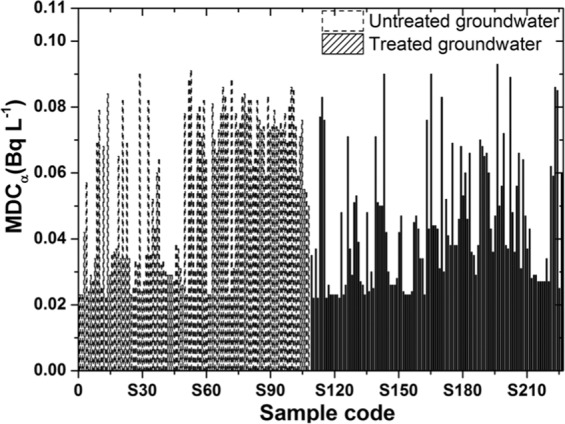
The minimum detectable concentration of gross alpha.

The minimum detectable concentrations (MDCs) of gross alpha and gross beta were calculated in Eq. (3). The highest MDC values of gross alpha were inconsistent and ranged from 0.022–0.091 Bq L^−1^ with average values of 0.057 Bq L^−1^ for the untreated groundwater and ranged from 0.022–0.093 Bq L^−1^ with average values of 0.044 Bq L^−1^ for the treated groundwater (Fig. 1).

**Figure 2:**
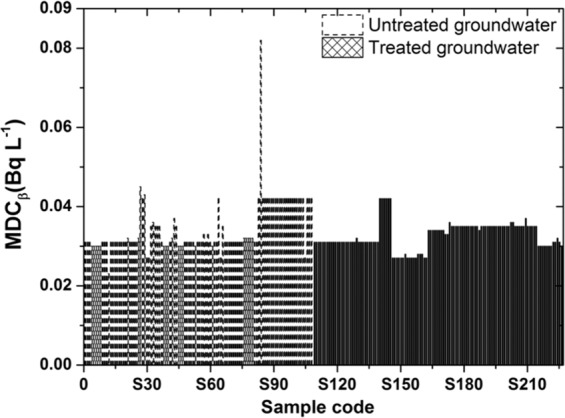
The minimum detectable concentration of gross beta.

In contrast, the MDC of the gross beta was relatively constant for the untreated groundwater in the range 0.023–0.081 Bq L^−1^ with average values of 0.034 Bq L^−1^ and for the treated groundwater in the range 0.027–0.042 Bq L^−1^ with average values of 0.033 Bq L^−1^ (Fig. 2).

The p-values of the mean comparison tests (t-tests) show that the difference is significant (p < 0.05) between the MDCs of the untreated and treated groundwater samples. The mean MDC value of the untreated groundwater samples is higher than that of the treated groundwater samples by approximately 1.2 times for gross alpha and 1.1 times for gross beta.

### Determination of gross alpha and beta activity concentrations

In the untreated groundwater samples, for 34 samples of the 108 analyzed samples, the overall gross alpha activity is higher than the MDC_α_; for 57 samples of the 108 analyzed samples, the gross beta activity is higher than the MDC_β_; 25 samples can be detected at both the gross alpha and gross beta activity concentrations.

In addition, in the 118 total treated groundwater samples, there are 37 samples whose overall gross alpha activities are higher than the MDC_α_, there are 64 samples whose gross beta activity is higher than the MDC_β_; and there are 31 samples whose gross alpha and gross beta activity concentrations could be recorded.

Table 1 presents the gross alpha and gross beta activity concentrations determined in the groundwater samples for both cases in this work. The median values of gross alpha and gross beta activities in untreated groundwater samples were 0.252 ± 0.042 Bq L^−1^ in the range of 0.026–0.748 Bq L^−1^ and 0.154± 0.015 Bq L^−1^ in the range 0.036–0.519 Bq L^−1^, respectively. In terms of the treated groundwater, the average values for the gross alpha and gross beta activities were 0.120 ± 0.026 Bq L^−1^ in the range 0.024–0.450 Bq L^−1^ and 0.152 ± 0.014 Bq L^−1^ in the range 0.027–0.632 Bq L^−1^, respectively. The p-values of the mean comparison tests (t-tests) show that the difference between the untreated and treated groundwater samples is significant (p < 0.05), and the mean gross alpha activity concentration for the untreated groundwater is approximately two times higher than that of the treated groundwater samples. However, the p-values of the mean gross beta activity concentration show that the similarity between untreated and treated groundwater is not significant (p > 0.05).

**Table 1:** Gross alpha and gross beta activity concentrations of the groundwater samples with uncertainties for both cases.

Untreated groundwater					Treated groundwater				
Sample code	Gross alpha		Gross beta		Sample code	Gross alpha		Gross beta	
	A_α_	ΔA_α_	A_β_	ΔA_β_		A_α_	ΔA_α_	A_β_	ΔA_β_
S1	0.060	0.014	—	—	S109	0.083	0.020	0.099	0.013
S2	0.071	0.015	—	—	S111	0.076	0.020	0.088	0.012
S8	0.058	0.016	0.092	0.012	S113	—	—	0.338	0.020
S9	—	—	0.047	0.010	S115	—	—	0.055	0.011
S12	0.403	0.063	0.234	0.017	S116	—	—	0.067	0.011
S14	—	—	0.042	0.010	S125	0.060	0.015	—	—
S15	0.039	0.013	—	—	S128	0.055	0.015	—	—
S19	0.074	0.027	—	—	S129	0.252	0.042	0.260	0.017
S21	0.521	0.078	0.344	0.020	S130	0.153	0.033	0.113	0.013
S23	—	—	0.118	0.013	S131	0.065	0.019	0.172	0.015
S26	0.092	0.018	—	—	S135	—	—	0.100	0.012
S29	0.464	0.073	0.314	0.022	S139	—	—	0.058	0.011
S31	0.029	0.011	—	—	S144	—	—	0.050	0.014
S32	—	—	0.183	0.016	S146	—	—	0.027	0.009
S33	0.382	0.061	0.245	0.018	S150	—	—	0.088	0.011
S34	0.068	0.015	0.043	0.012	S151	—	—	0.132	0.013
S35	0.183	0.033	0.096	0.013	S152	0.057	0.013	—	—
S36	—	—	0.037	0.011	S153	0.024	0.009	—	—
S37	0.287	0.049	0.457	0.021	S157	—	—	0.277	0.017
S38	—	—	0.036	0.010	S158	0.092	0.024	0.200	0.015
S39	—	—	0.149	0.014	S159	0.208	0.034	0.297	0.018
S40	—	—	0.175	0.015	S160	0.086	0.019	0.115	0.012
S41	0.144	0.024	0.069	0.012	S161	—	—	0.161	0.014
S42	0.162	0.026	0.051	0.011	S162	0.034	0.010	0.122	0.012
S43	0.593	0.060	0.119	0.015	S163	—	—	0.095	0.013
S44	0.493	0.053	0.117	0.014	S164	0.082	0.020	0.255	0.018
S46	0.052	0.017	0.178	0.015	S165	—	—	0.173	0.015
S47	0.053	0.017	—	—	S166	—	—	0.124	0.014
S48	0.026	0.010	—	—	S167	0.070	0.019	0.037	0.011
S50	0.081	0.031	0.084	0.012	S168	0.083	0.020	0.144	0.014
S52	—	—	0.374	0.020	S170	—	—	0.106	0.013
S54	0.097	0.028	0.204	0.015	S172			0.153	0.014
S57	0.748	0.097	0.393	0.021	S173	0.264	0.036	—	—
S59	0.722	0.095	0.519	0.025	S174	0.045	0.015	0.058	0.012
S60	0.266	0.048	0.296	0.019	S175	—	—	0.155	0.015
S63	—	—	0.111	0.013	S176	0.039	0.018	0.041	0.011
S64	—	—	0.053	0.014	S177	0.088	0.020	0.058	0.012
S66	—	—	0.058	0.012	S178	—	—	0.143	0.015
S69	0.086	0.033	0.057	0.011	S180	—	—	0.103	0.013
S76	0.366	0.062	0.228	0.016	S181	0.082	0.025	0.252	0.018
S77	0.462	0.073	0.385	0.021	S182	0.055	0.018	0.203	0.017
S78	0.420	0.070	0.155	0.014	S183	—	—	0.106	0.014
S79	0.388	0.065	0.176	0.015	S188	—	—	0.134	0.014
S80	0.457	0.072	0.157	0.014	S189	—	—	0.129	0.014
S81	0.162	0.043	0.210	0.016	S190	—	—	0.144	0.015
S82	0.070	0.025	—	—	S191	—	—	0.156	0.015
S83	—	—	0.046	0.016	S192	0.092	0.026	0.331	0.020
S84	—	—	0.194	0.018	S193	0.059	0.018	0.134	0.014
S85	—	—	0.210	0.018	S194	—	—	0.130	0.014
S86	—	—	0.224	0.019	S195	0.088	0.022	0.190	0.016
S87	—	—	0.215	0.019	S196	0.333	0.060	0.349	0.021
S88	—	—	0.201	0.018	S197	—	—	0.234	0.017
S89	—	—	0.183	0.018	S198	0.066	0.022	0.246	0.018
S90	—	—	0.070	0.014	S199	0.092	0.029	0.206	0.017
S91	—	—	0.061	0.014	S200	0.040	0.015	0.071	0.012
S92	—	—	0.045	0.014	S202	0.450	0.069	0.632	0.029
S93	—	—	0.048	0.014	S203	0.205	0.034	0.093	0.013
S94	—	—	0.043	0.013	S204	0.037	0.014	—	—
S96	—	—	0.057	0.014	S205	0.223	0.038	0.254	0.018
S97	—	—	0.057	0.014	S206	—	—	0.127	0.014
S98	—	—	0.076	0.014	S208	0.087	0.027	0.139	0.015
S99	—	—	0.076	0.014	S209	0.220	0.035	0.186	0.016
S100	—	—	0.055	0.014	S210	0.060	0.017	0.052	0.012
S101	—	—	0.051	0.014	S211	—	—	0.044	0.011
S104	—	—	0.089	0.015	S218	—	—	0.032	0.010
S108	—	—	0.070	0.014	S221	—	—	0.103	0.013
					S222	—	—	0.036	0.010
					S223	—	—	0.183	0.015
					S224	0.318	0.061	0.324	0.019
					S226	—	—	0.051	0.011
**Mean**	**0.252**	**0.042**	**0.154**	**0.015**	**Mean**	**0.120**	**0.026**	**0.152**	**0.014**
*Range	**0.026–0.748**		**0.036–0.519**		*Range	**0.024–0.450**		**0.027–0.632**	

*Range: min – max.

—: not detectable.

There are 19 groundwater samples whose gross alpha activity concentrations are higher than the recommended upper limit value^1,2^ of 0.1 Bq L^−1^, and there are 4 samples whose gross alpha activity concentrations are higher than the recommended value of 0.5 Bq L^−1^ WHO and IAEA^3,4^. After treatment processing, there are only 9 groundwater samples whose gross alpha activity concentrations are higher than the recommended value^1,2^, and no detectable gross alpha activity concentration is higher than the recommended upper limit value^3,4^; all values of gross beta activity concentrations are lower than the recommended upper limit value^1–4^ of 1 Bq L^−1^ for both groundwater cases, as shown in Table 1.

In this study, the gross alpha and gross beta activity concentrations were combined between the untreated and treated groundwater samples, which were 0.183 ± 0.034 Bq L^−1^ in the range 0.024–0.748 Bq L^−1^ for the gross alpha activity concentration and 0.152 ± 0.015 Bq L^−1^ in the range 0.027–0.632 Bq L^−1^ for the gross beta activity concentration. The results were compared with results obtained from samples that were collected in various regions or countries in similar studies, as shown in Table 2. The mean gross alpha and gross beta activity concentrations were measured in the range of previously published values.

**Table 2:** Gross alpha and gross beta activity concentrations in some regions or countries.

Region/Country	A_α_ (Bq L^−1^)		A_β_ (Bq L^−1^)		References
	Mean	Range	Mean	Range	
Albania	—	0.01–0.126	—	0.029–0.884	Cfarku^8^ *et al*.
Balaton/Hungary	0.189	0.035–1.749	0.209	0.033–2.105	Jobbágy^7^ *et al*.
Galati/Romania	0.022	<0.06–0.852	0.076	<0.025–0.435	Pintilie^9^ *et al*.
Hail/Saudi Arabia	2.150	0.170–5.140	2.600	0.480–5.160	Shabana and Kinsara^26^
Italy	—	<0.008–0.186	—	<0.048–0.150	Forte^27^ *et al*.
Katsina/Nigeria	—	0.080–2.300	—	0.120–4.970	Muhammad^28^ *et al*.
Nevsehir/Turkey	0.192	0.080–0.380	0.579	0.120–3.470	Turhan^19^ *et al*.
Sao Paulo and Minas Gerais States/Brazil	—	0.001–0.428	—	0.120–0.860	Bonotto^6^ *et al*.
Sebia	—	0.001–0.013	—	0.041–0.173	Janković^29^ *et al*.
Southern/Vietnam	0.183	0.024–0.748	0.152	0.027–0.632	Present study

### Evaluation of the annual effective dose for ingestion the groundwater samples

The origin of the gross alpha and gross beta activity concentrations was not investigated in this study. Gross alpha activity in groundwater is mainly due to uranium and its progenies, such as Po-210, Ra-226, and occasionally Th-232. The gross beta activity concentrations^6–9^ are probably mainly caused by K-40, Pb-210, and Ra-228. The 25 untreated and 31 treated groundwater samples detected both gross alpha and gross beta activities, and the results were used to calculate Pearson’s correlation coefficient to estimate the relationship between emitted radionuclides of gross alpha and gross beta. The strength of the correlation is based on the guide that Evans (1996) suggested for the absolute value of r: 0–0.19 corresponds to a very weak correlation, 0.20–0.39 corresponds to a weak correlation, 0.40–0.59 corresponds to a moderate correlation, 0.60–0.79 corresponds to a strong correlation, and 0.80–1.0 corresponds to a very strong correlation; a minus constitutes a negative correlation, and a plus constitutes a positive correlation^25^.

**Figure 3:**
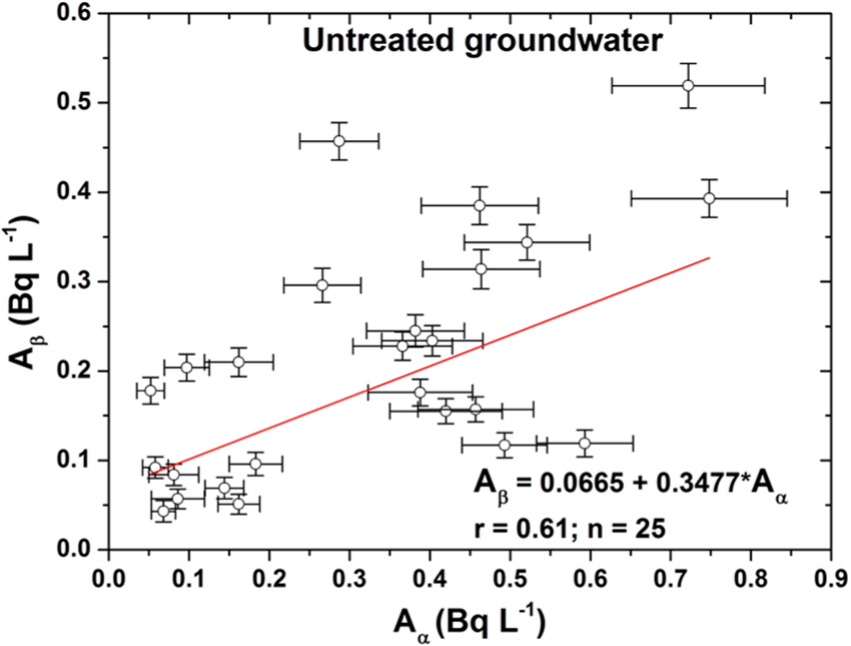
The correlation function of the gross alpha and gross beta activity concentrations for the untreated groundwater samples.

**Figure 4:**
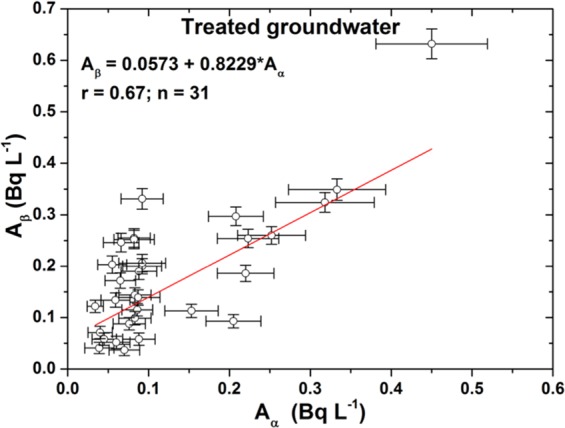
The correlation function of the gross alpha and gross beta activity concentrations for the treated groundwater samples.

The results are presented in Figs. 3 and 4, and they show that Pearson’s correlation coefficient between the gross alpha and gross beta for the untreated groundwater samples is 0.61 and that of the treated groundwater samples is 0.67. The results showed that if the existing original radionuclides emitted only beta particles such as Sr-90, Y-90, Ba-133, and Cs-137 in the samples, it was difficult to obtain a strong positive correlation. Hence, it follows that among the radionuclides, the major sources of beta radiation are the uranium and thorium decay series radionuclides.

**Figure 5:**
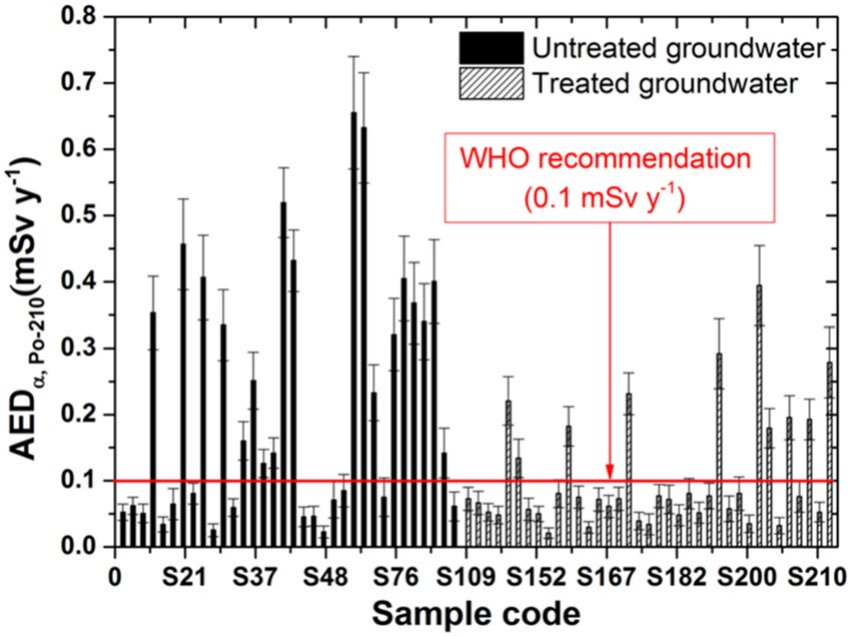
The annual effective dose due to intake of Po-210 in the groundwater samples.

**Figure 6:**
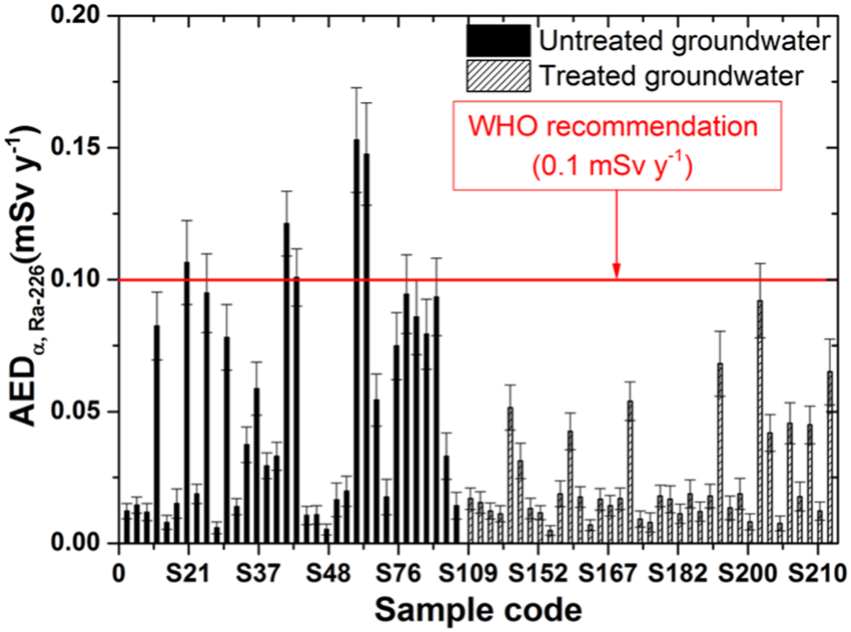
The annual effective dose due to intake of Ra-226 in the groundwater samples.

**Figure 7:**
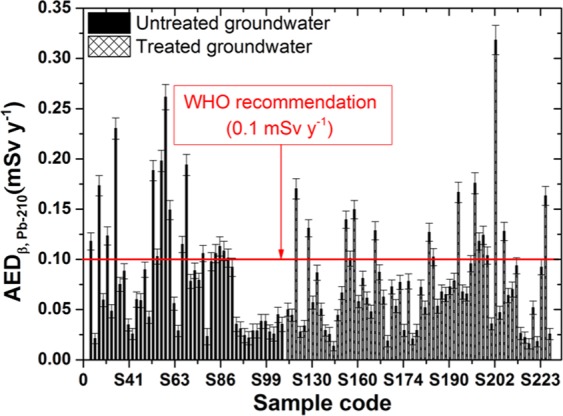
The annual effective dose due to intake of Pb-210 in the groundwater samples.

**Figure 8:**
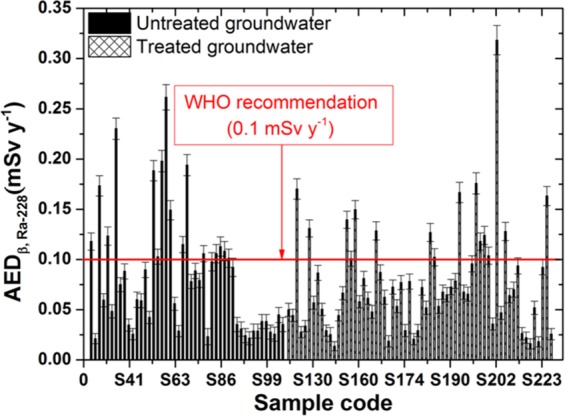
The annual effective dose due to intake of Ra-228 in the groundwater samples.

Moreover, the gross beta activity concentrations from K-40, which is negligible because of the age-dependent effective dose conversion factor for the adult person (CF_K-40_ = 6.2 × 10^−6^ mSv Bq^−1^), are the smallest of the other radionuclides. Therefore, to calculate the annual effective dose, we considered the gross alpha activity to be due to Po-210 and Ra-226 and the gross beta activity to be due to Pb-210 and Ra-228, which were radionuclide emitters with the highest age-dependent effective dose conversion factor^9,24^. The annual effective doses (mSv y^−1^) for the adult age group in southern Vietnam due to intake of Po-210, Ra-226, Pb-210, and Ra-228 from groundwater samples are represented in Figs. 5–8.

For the untreated samples, the annual effective doses for alpha emitters from Po-210 and Ra-226 radionuclides were calculated with ranges between 0.023–0.655 mSv y^−1^ and 0.005–0.153 mSv y^−1^, with mean values of 0.221 ± 0.037 mSv y^−1^ and 0.052 ± 0.009 mSv y^−1^, respectively. In addition, for the treated groundwater samples, the annual effective doses for alpha emitters from Po-210 and Ra-226 radionuclides were calculated with ranges between 0.021–0.394 mSv y^−1^ and 0.005–0.092 mSv y^−1^, with mean values of 0.105 ± 0.023 mSv y^−1^ and 0.024 ± 0.005 mSv y^−1^, respectively (Table 3).

**Table 3:** The annual effective dose for some radionuclides through ingestion of the groundwater samples.

Radionuclides	Untreated groundwater (mSv y^−1^)		Treated groundwater (mSv y^−1^)	
	Mean	Range	Mean	Range
Po-210	0.221 ± 0.037	0.023–0.655	0.105 ± 0.023	0.021–0.394
Ra-226	0.053 ± 0.009	0.005–0.153	0.024 ± 0.005	0.005–0.092
Pb-210	0.077 ± 0.008	0.018–0.261	0.077 ± 0.007	0.014–0.318
Ra-228	0.077 ± 0.008	0.018–0.261	0.077 ± 0.007	0.014–0.318
WHO and IAEA^3,4^	0.1			

For the untreated samples, Figs. 5, 6 show that the annual effective doses of 19 samples for Po-210 and 5 samples for Ra-226 are higher than the recommended 0.1 mSv y^−1^ by WHO and IAEA^3,4^. However, for the treated groundwater samples, the annual effective dose results are reduced in up to 10 samples for Po-210 and are not detectable in samples for Ra-226. Moreover, for the untreated samples, the mean value of the annual effective dose of the Po-210 radionuclide is 2 times higher and up to 6.5 times than that of the reference value, which is the highest for one adult person. Fortunately, the results are reduced by a factor of two for both radionuclides, which means that the treatment process is effective in removing the original radionuclides, which emit alpha particles.

Because the age-dependent effective dose conversion factors for Pb-210 and Ra-228 are equal, the results for both radionuclides are the same and are represented in Figs. 7, 8. The annual effective doses are calculated with ranges between 0.018–0.261 mSv y^−1^ and 0.014–0.318 mSv y^−1^, with mean values of 0.077 ± 0.008 mSv y^−1^ and 0.077 ± 0.007 mSv y^−1^ for the untreated and treated groundwater samples, respectively. The results in this work are lower than the recommended values by WHO and IAEA^3,4^ for both radionuclides.

## Conclusions

In the present work, the annual effective dose of the groundwater samples was evaluated to assess its compliance with national and international regulations. Therefore, this work can be used as a baseline for ascertaining possible changes in environmental radioactivity due to industrial and other human activities. The gross alpha and gross beta activity concentrations were determined for 108 untreated groundwater and 118 treated groundwater samples, which were collected in southern Vietnam.

In most cases, the gross alpha and gross beta activity concentrations were below the recommended values. The results were combined between the two kinds of groundwater samples to allow for comparisons with various regions or countries in similar studies, and the results show that the mean gross alpha and gross beta activity concentrations were measured in the acceptable range.

The annual effective dose was based on the assumption that major contributions of radionuclides were evaluated due to the ingestion of drinking water. The annual effective doses for Pb-210, Ra-226, and Ra-228 radionuclides are lower than those recommended reference values established by WHO and IAEA, except for Po-210.

Further investigation is required to estimate the annual effective dose due to the alpha- and beta-emitting radionuclides in the water samples. It is necessary to determine the specific activity of all radionuclides in the samples with the highest age-dependent effective dose conversion factor; in addition, the annual effective dose does not exceed 0.1 mSv y^−1^.
